# Xeno kidney: revolutionizing kidney disease treatment

**DOI:** 10.3389/fneph.2026.1707170

**Published:** 2026-02-20

**Authors:** Diksha Makkar, Diksha Gakhar, Aruna Rakha

**Affiliations:** Department of Translational and Regenerative Medicine, Postgraduate Institute of Medical Education and Research (PGIMER), Chandigarh, India

**Keywords:** clinical trials, genetic modifications, immunosuppressive, kidney, nonhuman primates, transgenic animals, xenoantigen, xenotransplantation

## Abstract

The prevalence of end-stage kidney failure has been exponentially increasing, leading to a gross mismatch between the number of patients who may benefit from transplantation and the limited supply of suitable donor organs. As renal transplantation remains a viable and the most effective option for end-stage kidney disease, the fact remains that the availability of eligible human donor organs is highly unlikely to meet the projected demand. This undermines the need for alternative strategies, including therapies and the development of transplant substitutes. In this context, xenotransplantation has emerged as a lucrative avenue for patients with renal failure who struggle to obtain a suitable graft promptly. The pig is currently the most preferred animal donor for kidney due to its physiological analogy to humans. Nevertheless, xenotransplantation is associated with certain complications as well, which broadly include the risk of hyperacute rejection mediated by preexisting antibodies to xenogeneic antigens, the stimulation of innate immune responses, and thereby the possibility of chronic rejection. Recent advances in xenotransplantation research have offered hope in overcoming these roadblocks and transforming the field of nephrology in the coming years. Genetic engineering has enabled creating low-immunogenicity grafts from donor pigs, including GalTKO (lacking α-Gal epitopes/galactose-α-1,3-galactose knockout) and gene knockouts that limit the complement system activation and clot formation. Furthermore, advances in immunosuppressive regimens, such as co-stimulation blockade and anti-complement treatment, hold great promise for xenograft acceptance and long-term results. In addition, numerous strategies are being explored to induce tolerance, such as mixed chimerism or regulatory T-cell therapy, to achieve a condition of acceptable graft tolerance without dependency on lifelong immunosuppressive treatments. Collectively, these developments support the translational potential of xenotransplantation as a stand-alone treatment or as an adjunct to standard renal replacement therapies. Despite the setbacks, ongoing preclinical research and early clinical trials are expected to refine the safety, durability, and clinical applicability in a xenotransplantation setting.

## Graphical Abstract

**Figure:**
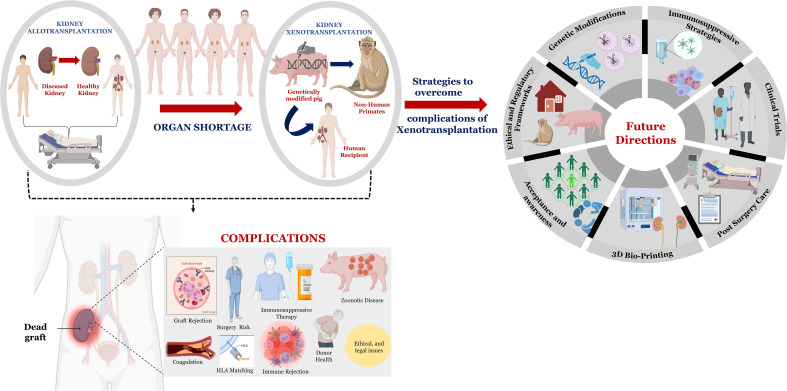

## 1 Introduction

Over 850 million individuals are affected by chronic kidney disease (CKD) globally. Estimates project that CKD will be the world’s fifth most common cause of death by 2040. Moreover, by 2100, it is foreseen to overtake all other causes of mortality (1, 2). CKD normally goes undetected until it progresses to end-stage kidney disease (ESKD) (3). Patients with ESKD who experience severe symptoms necessitate undergoing lifelong treatments, typically through either dialysis or transplantation. While dialysis extends survival, individuals undergoing dialysis generally experience inferior outcomes, a diminished quality of life, and heightened costs compared with those who undergo transplantation (4, 5).

As renal transplantation is the most feasible solution for ESKD, the emphasis lies on an urgent supply of kidneys that are readily available for transplantation; however, this too presents the persistent problem of immune incompatibility. Xenotransplantation represents a viable alternative, at least one that caters to the supply shortage. The present shortage of human organs could potentially be addressed or even eradicated by a never-ending organ supply from genetically engineered animals. It is likely to reduce the waiting time for transplants significantly, thereby giving patients a beacon of optimism during the need for life-saving surgeries.

The World Health Organization (WHO) defines xenotransplantation as “any procedure involving the transplantation, implantation, or infusion into a human recipient immune incompatibility of either: i) live cells, tissues, or organs from a non-human animal source or ii) human body fluids, cells, tissues, or organs that had *ex vivo* contact with live non-human animal cells, tissues, or organs.” However, it is crucial to remember that, before xenotransplantation can be extensively used as a remedy for organ shortages, the major incompatibility, ethical, scientific, and regulatory obstacles need to be thoroughly addressed.

Renal xenotransplant human trials are imminent due to the exponential increase in interest and the tremendous advancements made toward the practical application of xenotransplantation. Furthermore, with regard to an ideal donor species, pigs are preferred due to their large organ size and physiology, their rapid rate of reproduction, and their capacity for genetic alterations to correct molecular incompatibilities and reduce rejection (6). Renal grafts that prolong survival in non-human primates (NHPs) for weeks or months have been proven by preclinical studies, supporting the viability of kidney xenotransplantation from pigs (7, 8).

This review concisely communicates insights on combating immune rejection responses in xenotransplantation, summarizes the use of genetically modified pigs, and discusses the current survival longevity of xenografts in pig-to-NHP models. We also dive into the limited human trials that have been undertaken and the uncertainty they have revealed. Furthermore, we investigate previously ignored views that could improve our understanding of clinical outcomes with genetically modified animals and propose alternative methodologies for xenotransplant research (**Graphical Abstract**).

## 2 Literature search strategy and study selection

A comprehensive literature review was conducted including relevant studies on kidney xenotransplantation using databases such as PubMed, Scopus, and Google Scholar. The included articles were published from 1902 to 2025. The search terms were: kidney xenotransplantation, genetically modified pigs, nonhuman primates, and clinical xenotransplantation. All original articles, preclinical studies, clinical reports, relevant review articles, and latest news reports were included in this review.

## 3 Milestones in kidney xenotransplantation

**Figure 1:**
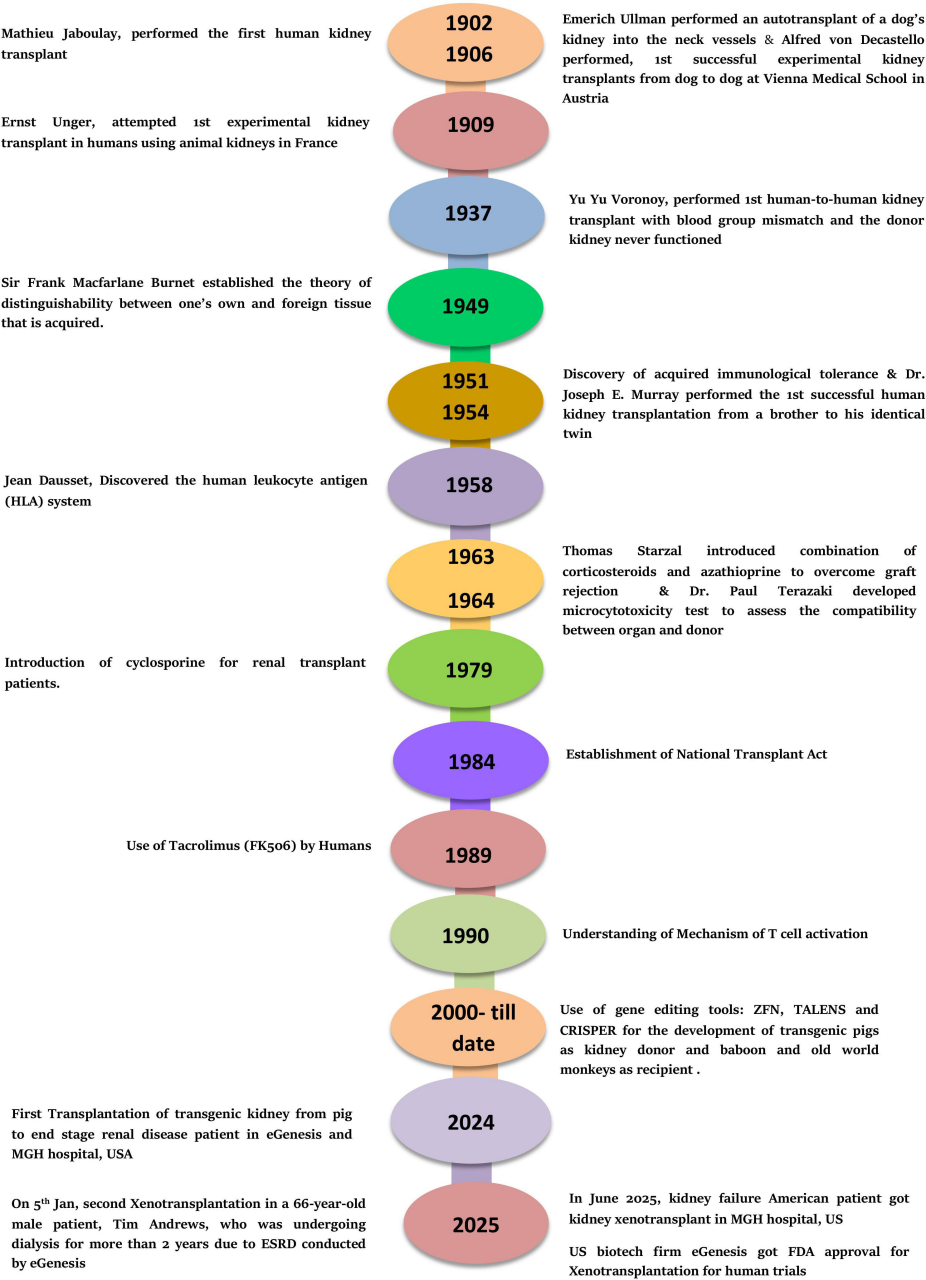
Milestones in kidney xenotransplantation.

Since the first kidney xenotransplantation in 1906 (9), significant advancements have been made, nearly eliminating the risks of hyperacute rejection (HAR). There have been substantial developments in critical areas of xenotransplantation, along with advancements in immunological theories and techniques (**Figure 1**).

## 4 Physiological and immune incompatibility in xenotransplantation

There has been limited translational success with NHP donor transplants conducted in the mid-1900s (10). However, it was soon understood that it would be impractical to obtain a sufficient number of organs from NHPs. Therefore, pigs were identified as the optimal choice due to their abundant availability, their anatomical and physiological similarities to humans, and the reduced risk of zoonotic diseases. On the other hand, the greater evolutionary distance between pigs and humans introduces additional biological hurdles that must be addressed for successful clinical translation. Overtime, several kidney transplants have been conducted from genetically modified porcine donors into NHPs such as Old World monkeys (OWMs). Although these porcine donors have significantly helped in dissecting molecular incompatibilities in xenotransplantation, there remains a gap that makes them not completely perfect for clinical applications (11–13). Rather than acting as isolated limitations, the major constraints are interconnected, and these connections and the strategies to overcome them are discussed in the review.

### 4.1 Donor size

Donors are typically sourced from commercial pig breeds. These pigs have heart and kidney sizes that are larger than required for human transplantation. This is resolved by using miniature pigs or by employing techniques that include the knockout (KO) of the growth hormone receptor. However, the latter approach results in systemic phenotypes similar to Laron syndrome, including a shorter stature with small bones, obesity, juvenile hypoglycemia, reduced fertility, reduced cancer incidence, a longer life span, an increased insulin sensitivity, and disproportionately smaller livers and kidneys (14). Thus, the right sizing must be balanced as it has a direct influence on graft performance.

### 4.2 Xenoantigens

There are substantial molecular differences between pigs and humans, predominantly in the setting of xenotransplantation. Xenoantigens, the inflammation pathways, and various components of the complement system remain central immunological barriers. The major leap came from the identification of three key porcine antigens, i.e., α-Gal, *N*-glycolylneuraminic acid (Neu5Gc), and Sd(a), and linking these to rapid graft injury. This led to stepwise genetic engineering in the donor: firstly, *GGTA1* KO (to remove α-Gal) and, later, additional KOs targeting *CMAH* (Neu5Gc) and *B4GALNT2* [Sd(a)] (15). These modifications substantially improved the graft survival in primates by reducing antibody binding. Notably, a study demonstrated that KO of the *CMAH*, *GGTA1*, and *β4GALNT2* genes in pigs resulted in organs that showed a negative crossmatch for numerous kidney transplant individuals. Interestingly, the *GGTA1* and *CMAH* genes are pseudogenes, resulting in the absence of the α-Gal and Neu5Gc epitopes in humans. However, post-birth, humans develop antibodies against α-Gal and Neu5Gc due to exposure to molecular mimics of these antigens. Humans have low levels of Sd(a)-reactive antibodies. In comparison to humans, OWMs have pre-formed natural antibodies (Nabs) against α-Gal and Sd(a); however, unlike humans, they do not have pre-formed antibodies against Neu5Gc as they retain a functional *CMAH* gene (16–19). As a result, triple-knockout (TKO) pigs paradoxically display increased immunoglobulin G (IgG) and IgM reactivity compared with *GGTA1*/*B4GALNT2* KO pigs. Consequently, this highlights that the ideal pig model for NHP studies differs from that needed for human clinical trials (20).

In addition to the carbohydrate epitopes discussed above, protein-level xenograft antigens shape the immune responses at the cellular level. Swine leukocyte antigens (SLAs) such as pig SLA class I and III molecules can cross-react with human leukocyte antigen (HLA) class I and II responses, respectively (21). It has been demonstrated that pigs with triple-gene (*GGTA1*, *B2M*, and *CMAH*) modifications expressed the SLA I^low^ phenotype, which influences the susceptibility to human immune responses (22). Several *in vitro* experiments demonstrated that the expression of SLA-1 increases with human tumor necrosis factor alpha (TNF-α), while human interleukin 17 (IL-17) decreases the TNF-α-mediated SLA-1 upregulation (23). Therefore, the SLA silencing strategy could be appropriate for the prevention of xenogeneic cellular immune responses.

### 4.3 Acute humoral rejection

Conventional immunosuppression inhibits the T-cell-mediated adaptive response, extending renal graft survival by days or weeks; however, grafts may later fail through acute humoral xenograft rejection (AXHR) (24, 25). AXHR, also known as acute vascular rejection or delayed xenograft rejection, is characterized by varying degrees of antibody and complement deposition, interstitial hemorrhage, microvascular thrombosis, endothelial cell changes, focal ischemic necrosis, and leukocytic infiltration. Even small amounts of anti-graft antibodies can initiate a self-amplifying loop of coagulation and inflammation (26). This clearly points that simply reducing the antibodies is mostly insufficient, which calls for effective methods to stabilize the vascular compartments and interrupt the thrombo-inflammatory reactions.

Taken together, these studies emphasize that donor engineering must target both humoral immunity and cellular activation triggers and not merely one or the other.

### 4.4 Other enzymes/proteins

The latest investigations in pigs with KO modifications targeting the Gal, NeuGc, and Sd(a) antigens exhibited transplant-induced coagulopathies to represent a significant obstacle to successful xenotransplantation. Several proteins and enzymes that show molecular incompatibilities between pigs and humans have been known, especially within the coagulation system.

For instance, pig thrombomodulin binds to human thrombin and is unable to activate protein C, resulting in graft thrombosis (27). These mechanistic insights underpin the addition of human transgenes (HTGs) such as human thrombomodulin (hTBM), CD39, and the tissue factor pathway inhibitor (TFPI) components. Mohiuddin et al. demonstrated that the combination of antibody therapy with the expression of hTBM has been instrumental to preventing humoral rejection and coagulation dysregulation, significantly lengthening graft survival to over 900 days in pig-to-baboon heart transplants (28).

An additional potential target for genetic modification is the endothelial protein C receptor (EPCR). While the pig EPCR is compatible with human protein C, Iwase et al. discovered a significant positive correlation between reduced human platelet aggregation and the expression of human EPCR in pig aortic endothelial cells (29). Furthermore, Wheeler et al. demonstrated that the expression of human CD39, which hydrolyzes ATP and ADP to impede thrombus formation, effectively prevented myocardial ischemia/reperfusion injury in transgenic pigs. Another prominent mismatch is the pig’s TFPI, which inhibits the blood coagulation process, resulting in increased thrombosis (30).

The key synthesis across these findings points that the genetic edits that target immunosuppression must be accompanied by vascular homeostasis mirroring human system processes; otherwise, the graft survives the initial immune hit, only to fail through dysregulated thromoboinflammation.

### 4.5 Porcine endogenous retrovirus

Finally, another barrier for xenotransplantation carries a distinct risk of cross-species transmission of pathogens. Swine viruses implicated in transplant outcomes include: i) those infecting healthy humans; ii) those affecting human transplant recipients; iii) those identical to the ones found in human transplant patients; and iv) those unique to swine. The third group includes porcine cytomegalovirus (PCMV) and porcine adenovirus, which has been implicated in issues related to pig and NHP transplant recipients. For example, PCMV has been associated with hematuria, decreased graft survival in pig-to-baboon transplants, and disseminated intravascular coagulation.

In parallel, other related γ-retroviruses such as koala retrovirus (KoRV), feline leukemia virus (FeLV), murine leukemia virus (MuLV), and porcine endogenous retroviruses (PERVs) raise unique concerns and can cause severe diseases including immunodeficiencies and tumors in their hosts, raising concerns about their transmission to humans (31, 32).

The three main PERV classes (A, B, and C) differ in their host tropism. While both PERV-A and PERV-B are present in all species of pigs and can infect human cells, PERV-C is harbored only by some species that infect pig cells, but can recombine with PERV-A to generate PERV-A/C variants with higher replicative capacity in human cells (33, 34). Different pig breeds have variable quantities of PERV proviruses, ranging from one to more than 100 copies (35). As a result, removal of all PERV proviruses from the pig genome is a difficult task. However, the screening and selection of donor pigs by excluding PERV-C, along with continued development of genetic or herd management approaches, appears to be the best strategy to reducing risks.

Conclusively, these studies highlight that the unsuitability of porcine donors is not driven by individual factors. Multiple factors that need co-optimization include the donor anatomy and physiology, immune responsiveness, the endothelial–coagulation axis, and rigorous pathogen control, which would be discussed in detail below.

## 5 Strategies to overcoming barriers

The most effective strategies for kidney xenotransplantation consist in layering complementary solutions rather than a single fix. Integrative platforms should combine: i) donor genetic edits; ii) tailored immunosuppression; iii) tolerance-oriented approaches; and iv) rigorous pathogen control.

### 5.1 Genetic modifications

Genetic engineering has a potential to transform a disastrous immunological event into a manageable event. Early pig-to-primate transplants failed largely due to the presence of pre-formed Nabs, leading to a high incidence of HAR. Deletion of the *GGTA1* gene (GalTKO) addressed the first barrier, directly supporting extended pig-to-baboon kidney transplants. This approach successfully spared HAR and markedly improved baboon survival, from 29 days with hCD55 transplants to 83 days (7, 36). The genetically modified kidneys when combined with tolerance strategies guided the subsequent work on two converging paths: firstly focusing on the removal of antigenic triggers and secondly on humanizing the graft vascular interface (37).

Despite preliminary success, in GalTKO pigs, xenografts were rejected afterward due to newly exposed targets of anti-non-Gal Nabs. The introduction of the CRISPR/Cas9 technology made it relatively easy and rapid to perform gene modifications. The University of Mississippi Medical Center (UMMC) and the University of Alabama at Birmingham (UAB) developed animals with 10 alterations (38). The first type of genetic modification consists of removing genes to eliminate the Nab targets, such as α-1,3-galactose (made by α-1,3-galactosyltransferase), Neu5Gc (produced by *CMAH*), and SDa (produced by *β4GALNT2*) (39–41). *In vitro* experiments with TKO pigs lacking *CMAH*, *Gal*, and *β4GALNT2* noticeably reduced Nab binding and improved the longevity in pig-to-cynomolgus macaque transplant models when paired with other transgenes (42). However, these outcomes also revealed a translational nuance: *CMAH* deletion could increase the antibody binding *vs*. GalTKO alone, implying that an antigen subtraction can cause amplification of the responses of default silent epitopes (42). Thus, genetic modification is an optimization strategy where the desired and non-desired consequences have to be monitored very closely.

Furthermore, extending upon the species-level incompatibilities, the major type of genetic alteration is based on dysregulation in the inflammatory pathways, coagulation, and complement that necessitates the addition of HTGs. As hitherto stated, the first genetically engineered pigs designed for xenotransplantation had human complement-regulating proteins such as CD55, with later versions incorporating CD46 (43), followed by the anticoagulant pathway components such as EPCR and TBM (44, 45). More recent alterations have focused mainly on anti-inflammatory proteins, such as anti-phagocytic proteins including the human macrophage inhibitory ligand CD47 (46) and heme-oxygenase (HO-1) (47). The development of the CRISPR/Cas9 gene editing technology now enables the mixing of these genetic modifications and the testing of various combinations aimed at system-level graft protection.

Despite these advances, breakthroughs in the field of xenotransplantation have sparked speculation about the success that might be achieved through increasingly sophisticated genetic modifications. However, this strategy further presents three major constraints. Firstly, there is a lack of consensus on the minimum number of edits: some are well established, such as GalTKO, while others rely solely on rodent models or are restricted to NHP studies due to exclusive research expenses (48). Secondly, despite the accuracy of modern gene editing tools in the integration of genes into genomes, consistent gene expression among transgenic animals remains a challenge. In parallel, tissue-specific gene expression is especially important as transgenes that are efficient in one tissue may not function properly in another. For example, whereas human CD47 expression in pig kidney transplants to baboons showed promise in preventing proteinuria, a high CD47 expression in renal tubular cells activated inflammatory responses via the CD47–TSP-1 pathway (48). Thirdly, while genetic alterations can reduce the innate immune responses to pig xenografts, they do not guarantee long-term rejection resistance. The variety of potential xenoantigens that may elicit adaptive immunological responses and antibody-mediated rejection (AMR) presents a substantial problem (48).

### 5.2 Immunosuppressive strategies

Before xenotransplantation can be turned into a clinical reality, the best immunosuppressive treatment without any unacceptable toxicity needs to be determined. It is widely acknowledged that xenotransplants are challenged by HAR, acute humoral rejection, and acute cellular rejection that can potentially be controlled by reducing the antibody burden, blocking T-cell activation, and suppressing the innate- and complement-mediated responsiveness (49–52).

The current therapeutics used in allotransplantation, such as anti-CD20 monoclonal antibody, mycophenolate mofetil (MMF), tacrolimus, anti-thymocyte globulin (ATG), rituximab, and corticosteroids, are often inadequate in xenotransplantation, unless intensified to levels that amplify infection risks (53, 54). This gap has driven strategic targeting of the xenogeneic immune response, in particular co-stimulation blockade.

Co-stimulation/blocking using an anti-CD40/anti-CD154 monoclonal antibody efficiently inhibited the T-cell-mediated antibody and cellular responses, especially in combination with conventional immunosuppressants (55). A study on a NHP model that compared a standard immunosuppressive regimen to one that included an anti-CD40 monoclonal antibody (mAb) resulted in considerably longer overall and rejection-free survival, without the development of thrombotic microangiopathy (TMA) or arterial vasculitis, as well as no indications of consumptive coagulopathy in the latter. These results support the hypothesis that inhibiting the indirect T-cell response is critical for preventing xenograft rejection and failure (56).

Building on these findings, the combination of T-cell depletion with CD40/CD154 co-stimulation pathway blockage has become a gold standard and has been repeatedly shown to improve survival in kidney xenotransplantation (57, 58). At the same time, anti-CD40/anti-CD154 monoclonal antibodies are not yet licensed by the US Food and Drug Administration (FDA) (59), which could delay approval of kidney xenotransplantation until thorough validation. To minimize dependence on a single unapproved mechanism target, anti-complement therapy and targeted anti-inflammatory strategies such as TNF-α or IL-6 blockade are being incorporated into therapy regimens (60). Nevertheless, phased trials are required in this area to conclusively determine their therapeutic advantages.

Furthermore, another method for inducing donor-specific immunological tolerance is simultaneous organ and hematopoietic stem cell transplantation (HSCT) in which a recipient receives both the donor and their hematopoietic cells by HSCT after non-myeloablative conditioning (61–63). Early investigations of pig-to-mouse transplant models showed that non-myeloablative conditioning regimens may produce mixed chimerism, implying that this strategy could promote tolerance across xenogeneic barriers in a rodent model (64). Mixed chimerism lowered not only HAR in rodent models but also the T-cell- and AMR in a pig-to-mouse paradigm (65). However, achieving mixed chimerism in preclinical pig-to-primate transplant models has proven difficult, with loss of the pig hematopoietic cells within 48 h likely due to preexisting antibodies (66, 67). Consequently, the use of hybrid strategies that utilize genetically modified pig kidneys in conjunction with continuous refining of immunosuppressive regimens is suggested in this field (68).

### 5.3 Vascularized thymic transplantation

Among the strategies to induce tolerance, direct transplanting the donor thymus targets the root of adaptive rejection by impeding the generation and selection of donor-reactive T cells. Sykes et al. demonstrated that transplantation of porcine thymic tissue into thymectomized mice led to the generation of mature mouse T cells that could tolerate porcine skin grafts largely through the deletion of donor-reactive T cells within the thymus (69). In order to avoid rejection of ischemic thymic grafts, scientists developed two methods for transplantation of vascularized thymic grafts in the late 1990s and the early 2000s: i) composite thymus + kidney (“thymokidney”) transplant (70) and ii) vascularized thymic lobe (VTL) transplant (71, 72). These vascularized thymic grafts were successful at inducing tolerance and promoting thymopoiesis in allogeneic swine kidney and heart transplant models (73–77).

In a pig-to-primate kidney xenotransplantation, GalTKO kidneys co-transplanted with thymus increased baboon survival from 29 days (36) to 83 days with donor-specific unresponsiveness (7). Contrary to xenograft sensitization, all baboon recipients developed a complication of severe nephrotic proteinuria as early as postoperative day (POD) 2 despite preserved renal function and low anti-pig antibody deposits (78). More recent studies have shed light on how thymic transplantation paired with targeted adjuncts (e.g., CTLA4–Ig and or additional edits such as hCD47) can prevent glomerular barrier dysfunction by monitoring proteinuria (46, 78, 79). These innovative techniques have resulted in the survival of many recipients of vascularized thymus and kidneys for up to 6 months (79, 80). The NYU group utilized thymokidneys on brain-dead patients with pig-to-human kidney transplants from September to November 2021, bridging the gap between NHP work and future clinical models.

Methodologically, thymokidneys are more easily manufactured and might become more popular for kidney xenotransplantation, while VTLs are more difficult to harvest and are susceptible to ischemia due to their intricate blood supply (7, 79, 80). Functionally, VTLs can theoretically offer more tolerance to any other organ graft compared with thymokidneys (77). Future studies should compare approaches for the production of thymic grafts, integrate thymic transplantation with mixed chimerism tolerance regimens, and explore the role of thymus co-transplantation in enhancing tolerance to other solid organs.

### 5.4 Zoonosis management

Even if the immunological and physiologic barriers are addressed, the major concerns regarding the potential spread of harmful zoonotic germs between species have hampered pig-to-human xenotransplantation efforts. PERVs are a focal example that are integrated into the pig genome as provirus DNA, which can be passed laterally to progeny (81, 82), making them qualitatively different from conventional exogenous infections.

A layered biosafety model may include selecting disease-free herds, housing pigs in germ-free amenities with stringent infectious disease screening procedures, isolating animals from outside sources, administering vaccinations, performing elective sterile C-sections for birth followed by the early weaning of piglets, and controlling the food sources.

Studies on patients with islet cell transplantation or *ex vivo* perfusion of pig livers and spleens found no evidence of PERV transmission, most likely due to the short exposure time and the lack of immunosuppressive therapy (83–85).

The absence of evidence in these settings, however, does not completely mitigate the risk associated with long-term implantation in those who are immunocompromised. Thereby treating PERV as a high-consequence hazard requires precise management either through genetic engineering or stringent donor screening (31).

In summary, the field is definitely moving toward clinically translational protocols while taking control of the late failure modes in the process.

## 6 Preclinical trials

Xenotransplantation holds a long history of using animal models such as mice, rats, and, most importantly, NHPs to examine the rejection mechanisms and to provide the most predictive platform for human translation (86, 87). Specifically regarding renal transplantation, pig-to-NHP transition studies have been critical due to their integration of immune incompatibilities, coagulation cascades, and clinically relevant immunosuppression in a way that smaller animal models cannot (88). Spanning over the past three decades, the survival outcomes have improved from 23 days with wild-type pig kidneys reported in 1989 (89) to several months in 2004 with pig kidney grafts from modifications such as hCD55 transgenic pigs in cynomolgus monkeys (8) and *GGTA1* deletion and the co-transplantation of vascularized thymic tissue from pigs to baboons (7).

A consistent theme across the preclinical literature is that survival of a stable kidney xenograft requires progress on two fronts in parallel: reducing the immunological triggers (especially pre-formed antibodies and xenoantigens) and stabilizing the post-transplant inflammation/coagulation pathways. Studies that introduced single HTGs to blunt immediate immune injury, such as complement regulation (e.g., hCD55), showed extension of survival into the ~90-day range in cynomolgus monkeys (8). Furthermore, the combination of genetic strategies (e.g., carbohydrate antigen deletion) with targeted immune interventions (including thymic tissue approaches and co-stimulation blockade) pushed the survival time further, but also highlighted how the outcomes can be ambiguous when anti-pig immunity differs between recipients at the basal level (7).

Through 2015, studies began to clarify the immunological risk associated with transplant outcomes. Higginbotham et al. demonstrated that rhesus macaques with low titers of anti-pig antibody achieved significantly longer survival (~130 days) following treatment with anti-CD154 mAb in comparison to high-titer recipients, emphasizing the significance of pre-transplant screening and personalized immunosuppression (90). At about the same time, in-parallel multi-gene donor engineering emerged to address coagulation and endothelial compatibility. A perfect example of this was pigs with combinations of complement regulatory and anti-inflammatory genes that support stable function for up to 260 days in baboons. The failures observed in these studies were mainly attributed to infections rather than immune rejection or coagulopathy (91), highlighting that, once immunomodulation takes control, infectious complications become the limiting factors.

A more in-depth analysis of immune compatibility reinforced that AMR is the main reason for late graft loss. In 2018, six rhesus monkeys with the least reactive crossmatches received kidney transplants from pigs deficient in *GGTA1* and *β4GalNT2*. Each animal received an immunosuppressive regimen based on anti-CD154 mAb. Three kidney xenografts were rejected within the first week, while the other kidney xenografts survived for 35, 100, and 435 days (92). This heterogeneity highlights that genetic engineering and potent immunosuppression can still be undermined by the recipient’s intrinsic immunity.

The field is progressing toward complex edits including deletion of the xenoantigen (e.g., *GGTA1*, *CMAH*, or *β4GalNT2*), the addition of human complement regulators, and the incorporation of anticoagulant/endothelial-protective genes. Kim et al. reported notable progress in the most recent trial on kidney transplantation from pigs to rhesus macaques by introducing xenoantigen deletion, careful recipient selection, and immune manipulation, which resulted in consistent survival over a year and the longest life span of a life-sustaining xenograft in a NHP (499 days) (13). These studies highlight the importance of identifying the incompatibilities that result in rejection episodes and combining them with manageable regimens to achieve long-term success (12, 13, 90–92).

A comprehensive analysis in 2020 described a detailed summary of xenotransplantation research where genetically modified pigs served as organ donors for Old World NHPs. The reactivity of NHP sera to *α1,3-galactosyltransferase* gene knockout (GTKO)/*β1,4-N-acetylgalactosaminyltransferase 2* knockout (β4GalNT2KO), GTKO, and TKO pig cells was evaluated in the study, which revealed that GTKO/β4GalNT2KO pigs have decreased antibody binding and cytotoxicity. An important point to note from these observations is its relevance in specific pathogen-free (SPF) environment (88). However, other studies, such as that of Yamamoto et al., in the same year reported an unexpectedly shorter TKO kidney pig graft survival with the insertion of HTGs [*CD46*/*EPCR*/*CD55*/*TBM*/*CD47*/heme oxygenase-1 (*HO1*)] compared with GTKO pig kidney (20). These observations underscore that different settings can interact in a complex manner that is dependent on the model tested and the baseline immune microenvironment and therefore particularly relevant in human translation studies.

In order to address AMR, early graft loss related to AMR was decreased with the use of anti-C5 as weekly injections for 70 days to temporarily suppress complement. Consequently, a median survival of 308 days was observed in five out of seven recipients who did not exhibit early AMR. All of the grafts, however, subsequently failed as a result of AMR (12). This study particularly reinforced that short-term complement control may delay, but not eliminate, the humoral rejection episodes. In a similar direction, the transplantation of TKO kidneys with multiple HTGs [complement-regulating genes (e.g., *CD46*, *CD55*, and *CD59*) and HLA-E, B2M, CD47, and PD-L1] into cynomolgus macaques suggested that long-term survival is achievable even without the stringent low-titer pre-screening (93). In contrast, limited kidney survival was observed by Ariyoshi et al., with 154 days in baboons transplanted with KO pig kidneys with a vascularized thymic graft along with multiple HTGs (*CD46*/*CD55*/*EPCR*/*TBM*/*CD47*/*HO1* or *CD39*/*EPCR*/*TBM*/*HO1*) (93), indicating AMR, infections, and systemic complications as persistent bottlenecks.

One of the most ambitious strategies in the context of genetic engineering extends beyond the classical carbohydrate antigen edits. With the most extensive genetic editing to date, Qin and his team edited 69 genes in live pigs for xenotransplantation. Out of these, 59 edits targeted porcine endogenous retrovirus (PERV) sequences to reduce the risk of cross-species viral transmission. The remaining 10 modifications addressed immunological and physiological barriers to graft survival, comprising three knockouts of major carbohydrate antigens associated with rejection and the insertion of seven human transgenes to support immune regulation, coagulation control, and long-term organ function. To support continued health of the transplanted organ, human genes were added in the final seven alterations. Over 20 cynomolgus macaques received kidney transplants from these genetically altered pigs, along with immunosuppressive medications. A total of 9 out of 15 monkeys that received kidneys with human gene editing survived longer than 50 days compared with those that did not. Of these monkeys, five lived longer than a year, while one lived longer than 2 years. The maximum survival was 758 days. The performance of the transplanted organs was comparable to that of native kidneys in these settings, supporting the idea that a broad genetic reconfiguration can yield longer and stable graft function, but under a precise validation (94).

A major recent advancement of utmost translational value included reproducible survival across multiple animals, clinically relevant immunosuppressants, and stringent monitoring protocols. A 2024 study demonstrated promising results with 10-gene-edited (10-GE) donor kidneys in baboon recipients, with successful long-term survival and consistent survival across numerous cases. These 10-GE alterations included the addition of human complement-regulating proteins and human anti-coagulant proteins, as well as the deletion of three known targets of pre-formed Nabs that are carbohydrate antigens. The survival of recipients with low levels of pre-formed anti-source pig antibodies were 337, 285, 278, 252, and 165 days. All recipients had an average survival of 220 days, with a median of 261 days. Perhaps the most exciting of all is that graft function was maintained for more than 6 months in four of the five low-risk recipients and more than 9 months in three of them. This work underscored the importance of effective immunosuppression in xenotransplantation by showing consistent long-term survival and sustained renal function in baboon recipients. Realizing long-lasting results without CD40/CD154 blocking significantly increases the probability that clinical kidney xenotransplantation will become feasible (95). At about the same time, another study in 2025 highlighted the generation of a donor pig with eight specific gene edits—*GTKO*/*CMAHKO*/*β4GalNT2KO*/*hCD46*/*hCD55*/*hCD59*/*hTBM*/*hCD39*—using the CRISPR/Cas9 system, the PiggyBac transposon system, and somatic cell cloning. The xenograft from these pigs survived for a mere 15 and 17 days in both NHPs, highlighting that even a sophisticated donor engineering can fail rapidly when humoral injury is not under control (96).

**Figure 2:**
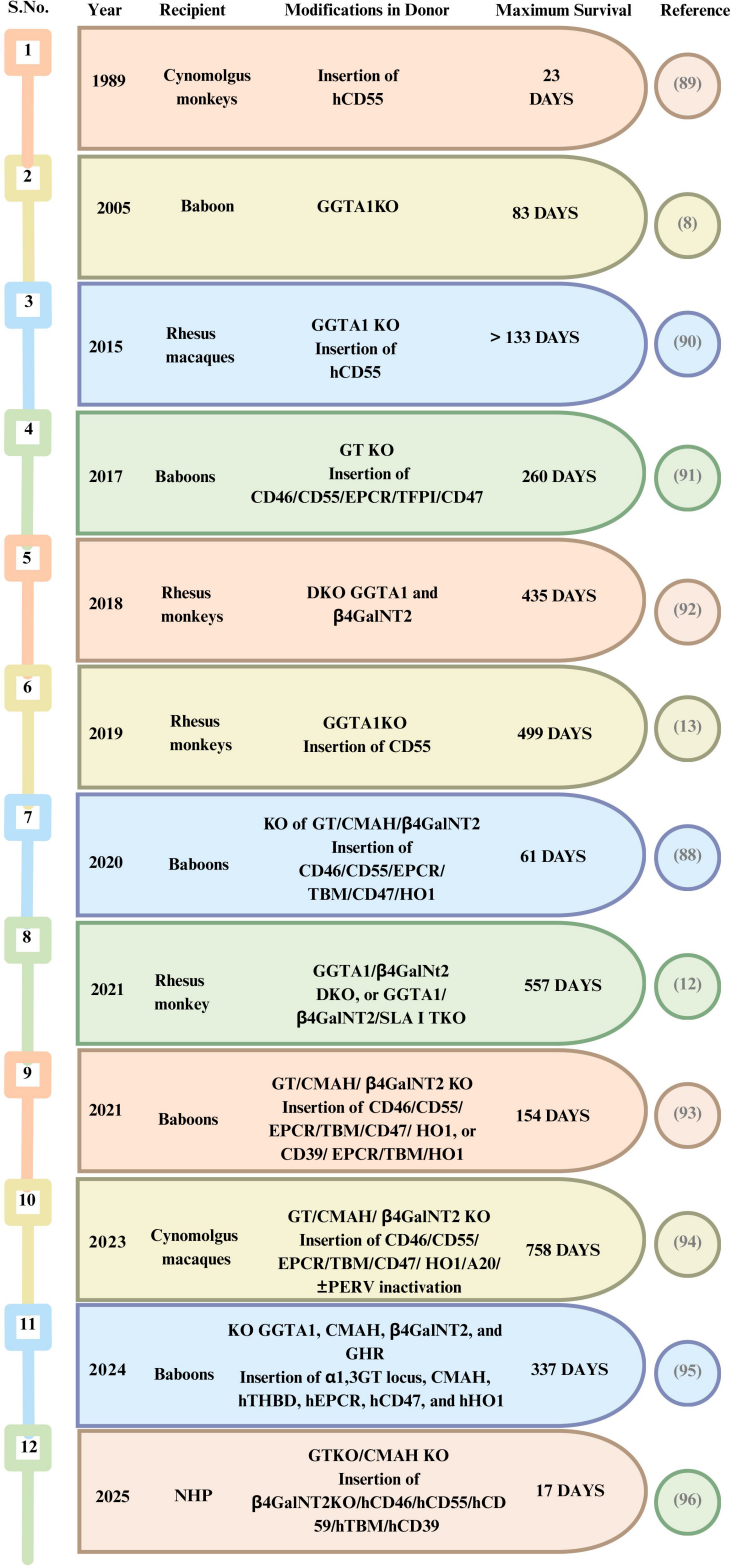
Highlights of kidney xenotransplantation in preclinical models—non-human primates.

Overall, while these preclinical studies have provided valuable information and breakthroughs in kidney xenotransplantation (**Figure 2**), many obstacles still remain before it can be considered practical for human use. These milestones are driven by remarkable improvements in donor genetics, recipient selection, and immunosuppressive regimens, but converging with foreseeable barriers, including AMR and infections, with a clear challenge to develop strategies that are both effective and clinically acceptable.

## 7 Clinical trials and human applications

**Figure 3:**
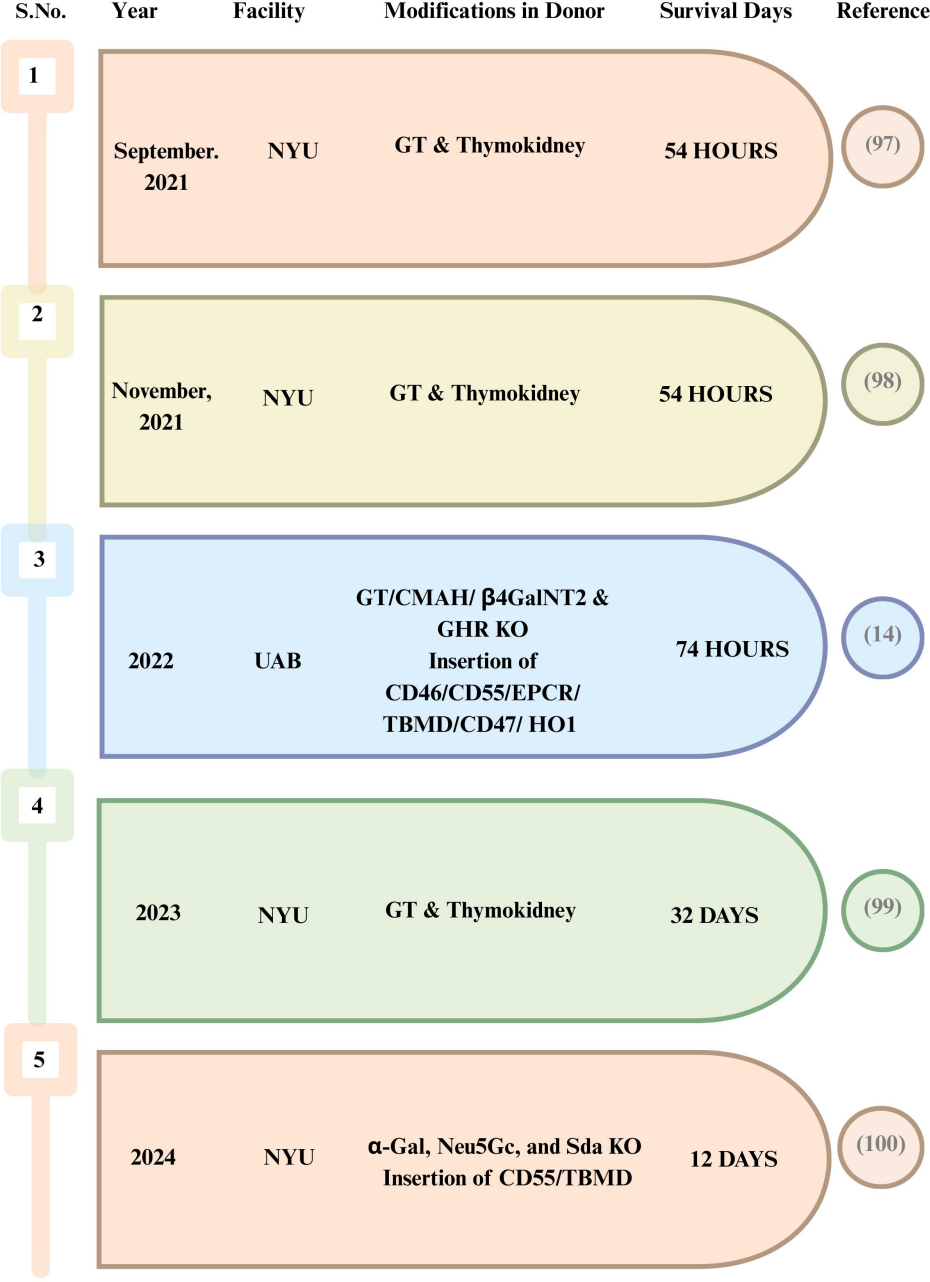
Highlights of kidney xenotransplantation in a brain-dead human recipient.

**Figure 4:**
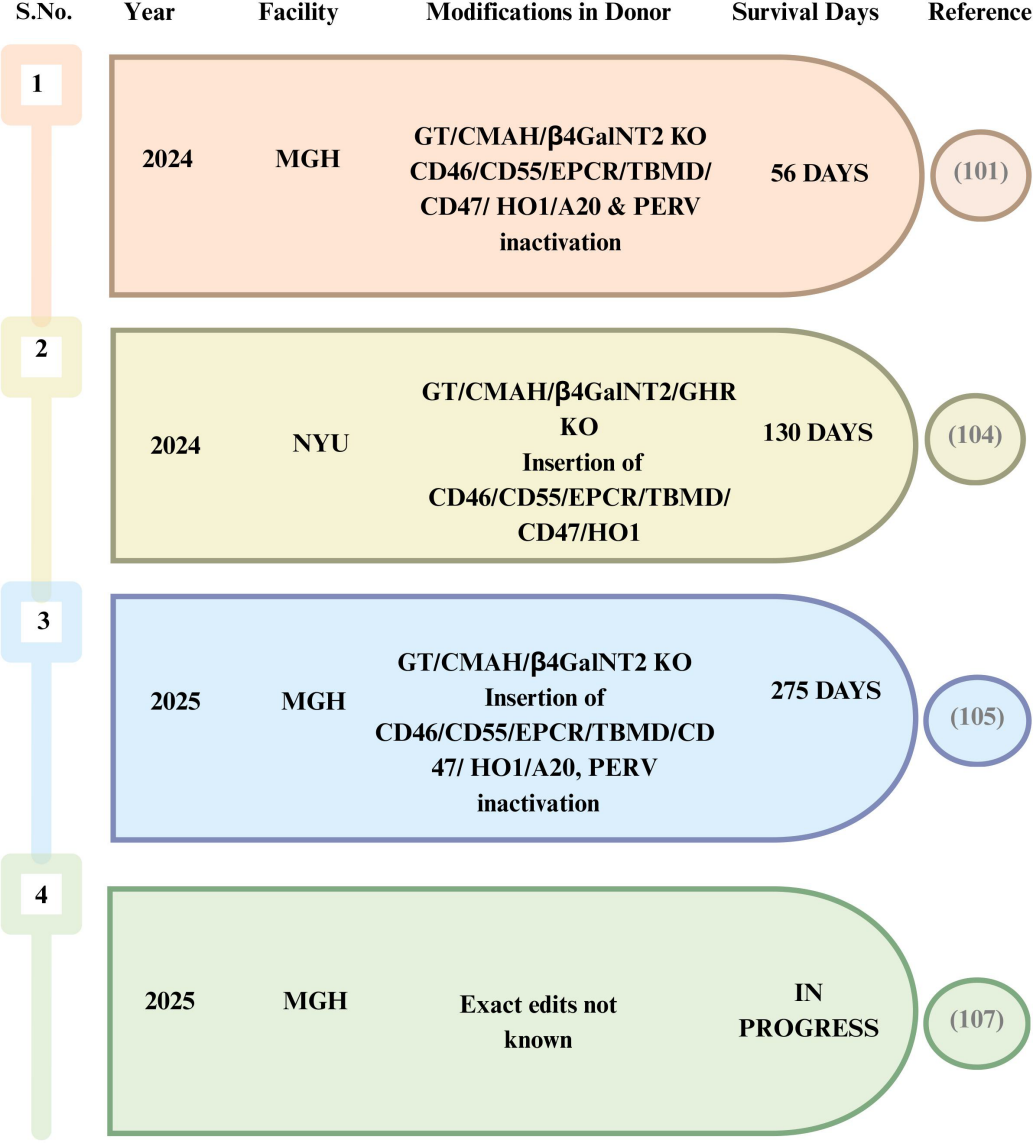
Highlights of kidney xenotransplantation in clinical models.

From 2023 to 2025, renal xenotransplantation has transitioned from a proof-of-concept research in brain-dead recipients (**Figure 3**) to a few limited experiences in living patients (**Figure 4**). Two notable clinical breakthroughs in 2021 further underlined the enthusiasm of starting clinical trials in NHP kidney xenotransplantation. The team of Dr. Montgomery carried out two xenotransplants from a pig to a brain-dead human recipient at NYU in 2021, with equal success (97). They utilized α-1,3-galactosyltransferase KO pigs and the kidney grafts were prepared 2 months before transplantation, with a porcine thymus placed beneath the renal capsule (74). Standard immunosuppressive protocols were followed, in addition to systemic anticoagulation via a heparin drip, with the total procedure lasting for 54 h (97, 98).

Another trial of a pig-to-brain-dead human kidney xenotransplantation was performed at the UAB (14). The genetic modifications included the removal of three relevant immunodominant xenoantigen carbohydrates: Neu5Gc, galactose-α-1,3-galactose, and Sd(a) blood group antigen. Moreover, the growth hormone receptor was knocked out to inhibit intrinsic xenograft growth. The kidneys were expressed with human CD46 and human decay-accelerating factor (hDAF) to prevent antibody-mediated complement injury. To further enhance thromboregulation, hTBM and human endothelial cell protein C receptor (hEPCR) were introduced to improve protein C activation and anticoagulant signaling. The immunosuppressive regimen followed a similar approach to that of allotransplantation and involved a continuous heparin infusion. After bilateral native nephrectomy, the right and left 10-GE pig kidneys were transplanted individually into the recipient. Unfortunately, this case did not provide positive outcomes. Over 3 days, the left kidney showed minimal urine output, while the right kidney showed a urine output of less than 1 L, which was followed by enhancements in the serum creatinine and blood urea nitrogen levels (more than 6 and 150 mg/dl. respectively). Histological examination performed 24 h after xenotransplantation revealed results attributed to the presence of TMA, despite efforts to reduce this consequence in the pigs through genetic changes and continuous heparin infusion.

In July 2023, surgeons at NYU transplanted an α-Gal KO pig kidney + pig thymus gland. The kidney and the thymus gland were procured from Revivicor Inc. (Blacksburg, VA, USA). The kidney functioned well even after 32 days in brain-dead patient, with survival on ventilator support (99).

By early 2024, xenotransplantation using a porcine donor with five gene edits (5-GE) was performed on a deceased human recipient in Xijing Hospital in China. Three glycan genes known to trigger HAR—*Neu5Gc*, *α-Gal*, and *Sda*—were knocked out, while the genes human CD55 (*hCD55*) and *hTBM* were inserted into the pig’s genome to alleviate uncontrolled activation of the complement and coagulation cascades with standard immunosuppression. The xenograft initially experienced delayed graft function in the first week, but the urine output improved. The single xenograft kidney maintained electrolyte and pH homeostasis from POD 12 to 19. However, AMR was persistent throughout the observation period of 22 days, with treatments such as intravenous immunoglobulin and plasma exchange mitigating the AMR. Activation of latent PCMV toward the end of the study was also observed, which might have contributed to the coagulation disorder in the recipient. This study had limitations, including the observation period lasting only 22 days and the use of the same lacunae that have already been reported in deceased patients (100).

At about the same time in March 2024, Massachusetts General Hospital (MGH) conducted the world’s first genetically modified pig kidney transplant into a living human on compassionate base use. Richard Slayman, a 62-year-old man with ESRD dependent on hemodialysis, received his kidney transplant from a CRISPR/Cas9 genetically modified pig on March 16, 2024 (101). Of the 69 genetic alterations, three genes that produce sugars on the surface of pig cells were eliminated. Moreover, seven human genes were added to generate proteins that help prevent organ rejection. Another 59 genetic alterations were applied to deactivate the viruses inserted into the pig genome (102). Simultaneously, they administered organs from these pigs with identical genomic alterations into cynomolgus macaques (*Macaca fascicularis*), with the macaques surviving for months to years. Transplantation to the patient appeared initially successful as the kidney began generating urine immediately following surgery and showed no signs of rejection. Unfortunately, the patient passed away 2 months later, which did not appear to be associated with his transplant as he was diagnosed with congestive heart failure a year before surgery (103).

NYU’s April 2024 experience illustrated another crucial bedside constraint that is less prominent in preclinical narratives: the surgeons successfully executed the first combined treatment that included a mechanical heart pump and a gene-edited pig kidney transplant in a 54-year-old lady suffering from both heart and kidney failure. The milestone was achieved in two phases: firstly, they implanted a mechanical heart pump and, 8 days later, performed the xenotransplant with a gene-edited pig kidney paired with a pig thymus gland (UThymoKidney) (104). However, recurrent hypotension limited the blood supply to the newly transplanted kidney, and there was no biopsy-based rejection. The kidney function eventually deteriorated and therefore removed, with a documented longest survival of 130 days. This case is a clear example of when a graft can fail due to intrinsic physiology despite immunological and vascular control.

In January 2025, MGH completed its second xenotransplantation in a 66-year-old male patient. Tim Andrews received a similarly engineered pig kidney with discharge within a week of transplantation. The graft continued to function properly, with serum creatinine of 1.2–1.4 mg/dl at 5 months after transplantation. He is the fourth individual in the world to have undergone genetically modified xenotransplantation (105). He lived with a pig kidney for 271 days, after which his body rejected the organ and was returned to dialysis. By January 2026, he is supposedly the first patient to receive human kidney after a pig kidney, as per news reports (106). Subsequently, in June 2025, MGH performed another xenotransplant in Bill Stewart, a 54-year-old athletic trainer patient. He is also reported to be doing well post-transplantation (107).

Considering the success in the transplants cited above, the US biotech firm eGenesis has received approval from the FDA to begin human trials of pig kidneys in September 2025 (108).

While these are small numbers in terms of success, the pattern is important. As the protocols stabilize and there is more accountability toward repeatability, along with in-parallel handling of the open challenges against post-transplant complications, we are definitely progressing toward fulfilling the prerequisites for formal clinical trials.

This field is now poised for structured human studies targeting optimization of the genetic changes, immunosuppressive drugs, and surgical procedures to enhance the results in patients in need. Besides these scientific and medical advances, the sector faces ethical, legal, and social challenges that may impede wider acceptance. The widespread adoption of such strategies depends upon trustworthy oversight and reproducible safety as much as on their technical success.

## 8 Ethical, legal, and social consequences

Even after tremendous genetic breakthroughs, xenotransplantation faces legal, regulatory, and ethical constraints to acceptance. Importantly, these issues are not only peripheral add-ons, but arise from the same concern of catering to the risks that can extend beyond an individual recipient. The possible risks reported have implications beyond many facets of clinical xenotransplantation research. These include concerns with the informed consent procedure, privacy rights, and the capacity of the study subjects to withdraw from studies requiring long-term infectious monitoring in order to safeguard the larger community, in parallel with scientific progress. These are issues that call for strenuous and continuing debate, and there are no easy answers to these (109). The ethical debates in xenotransplantation revolve around three domains: i) patient safety and autonomy; ii) animal wellbeing; and iii) population risk and societal trust. The discussion about these explains how improving upon one domain can undesirably affect the other.

### 8.1 Patient safety and informed consent

Ensuring patient safety and consent is a priority as patients not only face the conventional transplant-associated risks but also the susceptibility to PERV infections. In this regard, there have been growing ethical concerns and the responsibility to safeguard the general public from dangerous contagious diseases, which must be balanced against the necessity to discover innovative medical treatments for patients suffering from ESKD (110). Although genetic engineering and designated pathogen-free (DPF) facilities can substantially reduce the pathogen burden (31), the risks associated with other undetectable viruses, such as CMV or porcine roseolovirus, remain (111). Thus, the ethical challenge does not only imply a simple informed consent from the patient but also ensuring that the transplant recipient and the relatives understand the possible long-term implications (111), where a withdrawal from follow-up may be complicated when community health is at stake (112, 113). Furthermore, this challenge is amplified by the enforcement of legal rules such as quarantine provisions for contagious diseases, affecting close relationships (111).

### 8.2 Animal welfare

There are similar concerns with regard to the ethical treatment of the donor or recipient animals, which must be handled humanely and ensured that the genetic changes do not impair their health. Donor pigs normally receive better care than those in commercial piggeries as they are housed in extremely controlled environments and are subject to rigorous animal ethics legislation and committees (110). Furthermore, it is not only about pig welfare but also about reliance on NHPs for preclinical validations. In light of the proximity of their genetic makeup to that of humans, and thus the associated ethical boundaries in this regard, regulatory authorities such as the FDA need strict ethical compliance and special permissions for NHP research. To address these complications, experts need to propose solutions for ensuring humane living conditions and minimizing harm on the animals used in xenotransplantation, e.g., Rollin’s “minimum moral standing” (114).

### 8.3 Societal and public health impacts

Extending the discussion to include xenotransplant marketing, public perceptions and attitude about xenotransplantation, and the ethics involved, which are additional issues that must be addressed in detail, is necessary. Sufficient public education regarding the risks and advantages of xenotransplantation and the willingness to communicate on such issues are essential to earn trust in and acceptance of the procedure (114). Such complex ethical problems require constant surveillance and risk–benefit trade-offs, followed by emphasizing public engagement as a necessary ingredient for legitimacy, especially while using animals as organ donors (114).

### 8.4 Regulatory and legal considerations

Xenotransplantation increases the risks of zoonotic infections that are likely to have a public health dimension. Zoonotic invasion represents the most difficult element of its implementation as this necessitates the monitoring of patients and their close contacts throughout life, which is uncommon in other therapeutic areas (115). The regulatory regimens are characterized by a great deal of divergence in different countries, posing challenges to the concordance sought in order to promote safe and ethical xenotransplantation methods. Indeed, organizations such as the Council of Europe have emphasized the necessity of observations and follow-up work post-transplantation, which may entail the infringement of some rights of the patients in the interest of public wellbeing.

Concerns have been raised by regulatory organizations, such as the Council of Europe, with Article 21 of Recommendation Rec (2003)10 implying that patients may need to waive certain human rights to ensure close follow-up and monitoring. Regulatory and legal considerations must be addressed worldwide for the commencement and development of xenotransplantation. Individuals keen on xenotransplantation may travel to countries with more liberal policies in order to undergo the life-saving treatment, owing to the effects of globalization, differential healthcare, and regulatory laws across areas. This may increase the danger of zoonosis upon their return to countries with stricter animal husbandry and organ screening rules (116).

This emphasizes the importance of international consensus as a tool for reducing the risks that arise from differences in healthcare and other institutions. Without this convergence, the xenotransplantation field risks an environment where international inconsistency can shape a weak regulatory global risk.

### 8.5 Economic implementations

For the past two decades, xenotransplantation has been prominent in the public and the government perspectives. Nevertheless, aspects regarding the risk of infections and liability have caused the financial backing from the biotechnological sector to dwindle since the late 1990s.

Obtaining genetically edited source animals for animal-to-human organ transplant may involve several genetic engineering techniques, but this remains unattainable due to the difficulties associated with the technology and patenting. A further complication is the creation of a compliant source, which conforms to tough regulations such as lifetime surveillance and sample retention (109). Intellectual property and technical complexity further widen the barriers by limiting who can perform the large-scale production of compliant donor animals. Nevertheless, the long-term economic benefit is clear if xenotransplantation successfully reduces graft loss and reduces the dependence on dialysis. This could potentially generate large downstream savings and major quality of life gains (109).

## 9 Conclusion and future directions

As we consider the current advantages of kidney xenotransplantation as a medical technology, it is evident that implementing clinical translation is a real challenge. In the insolvable problem of the lack of organs, xenotransplantation offers humanity a view into a fairly optimistic future with a transformative solution. Despite the tremendous promise that the clinical xenotransplantation technology brings, efforts to move this technology from the laboratory bench to the bedside must be governed by robust ethical, regulatory, and safety frameworks with an unwavering focus on patient safety and the sanctity of science. The field is moving toward human application, and it is of utmost importance to evaluate the practicality and efficiency of xenotransplantation in everyday life.

The aspect of xenotransplantation is at the very cutting edge of development in medicine and promises to change transplant medications for the better sometime in the future. Genetic manipulation in donor pigs to reduce immunogenicity is not the only strategy being developed currently as many regenerative technologies and methods are also being explored to enhance xenograft endurance and performance. Such experimental methods include the use of bioengineering and modern 3D printing technology for individual organ scaffolds and enhancement of graft vascularization for better incorporation of the graft with its host and for durable function. Regenerative medicine techniques such as stem cell therapy and tissue engineering aim to repair and replace damaged tissues in an organ transplant and may, therefore, sustain the function and life span of the transplanted organ. Furthermore, advances in immunosuppressive therapy, such as targeted biologics and immunomodulatory drugs, are critical for inducing immunological tolerance and preventing rejection in xenotransplant recipients. If the anticipated upcoming clinical trials demonstrate consistent function and an acceptable safety, it can be considered as transformational medicine as a practical response to organ scarcity.

## 10 Limitations of review

This review provides a broad synthesis based on a comprehensive literature search. However, a formal systematic review framework was not employed; therefore, some relevant studies may have been inadvertently excluded. In addition, the rapidly evolving nature of kidney xenotransplantation research means that recently published or ongoing studies may not have been fully represented.
